# Double suture-button reconstruction for chronic volar dislocation of distal radioulnar joint: A case report

**DOI:** 10.1016/j.amsu.2022.104705

**Published:** 2022-09-17

**Authors:** Oryza Satria, Irsan Abubakar, Diaz Novera

**Affiliations:** a Department of Orthopaedic and Traumatology, Fatmawati General Hospital, Jakarta, Indonesia; b Division of Orthopaedic and Traumatology, Department of Surgery, Universitas Syiah Kuala, Zainoel Abidin General Hospital, Banda Aceh, Indonesia

**Keywords:** Distal radioulnar joint, Volar dislocation, Double suture-button reconstruction

## Abstract

**Introduction and importance:**

A distal radioulnar joint (DRUJ) dislocation is an uncommon occurrence. If not managed properly could result in severe functional impairment.

**Case Presentation:**

We report a case of a 45-year-old lady who was injured 6 months ago. She suffered a volar dislocation of the DRUJ and an ulnar head fracture. The DRUJ was stable after open reduction and reconstruction using suture-button technique.

**Discussion:**

Application of the first suture-button still result in positive ballottement with subluxation of ulnar head. Additional of second suture-button improved the stability and restored DRUJ motion.

**Conclusion:**

The double suture-button technique restored the DRUJ's stability and produced a good functional outcome.

## Highlights

• Distal radioulnar joint (DRUJ) dislocation is a rare occurrence accompanied by irreparable TFCC.
• Open reduction and reconstruction using double suture-button technique was performed as tendon graft carries high risk.
• This modified technique restored the stability, smooth motion and range of motion of DRUJ.

## 1 Introduction

A DRUJ dislocation is an uncommon occurrence. This injury, if misdiagnosed or mismanaged, causes a total loss of pronation-supination, resulting in a severe functional impairment [1]. Typically, a closed reduction with the forearm held in protonation is sufficient to treat an acute injury. Several surgical techniques have been proposed to address the problem of dorsal dislocation, however surgical correction of chronic volar dislocation is more challenging [2]. Here, we present the reconstruction of a chronic volar DRUJ dislocation with a partial ulnar head fracture and an impaction fracture of the distal radius using a double suture-button technique. This case report has been reported in line with the SCARE Criteria [3]

## 2 Presentation of case

**Fig. 1:**
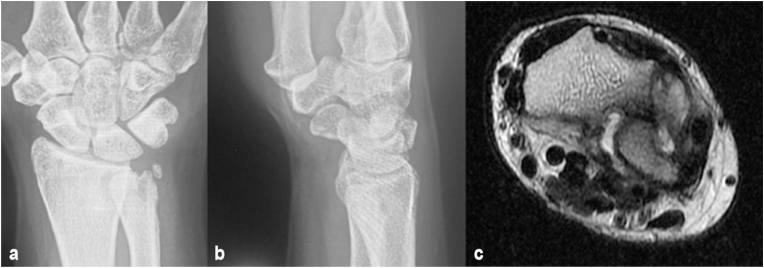
(a,b) Plain X-ray PA and Lateral showing ulnar head fracture and (c) axial MRI showing chronic volar dislocation with impacted fracture of volar-ulnar distal radius.

A 45-year-old right hand dominant woman injured her right wrist after falling while walking. She did not seek therapy for around six months before visiting a bone-setter. After nine months, she was unable to rotate her wrist and went an orthopedic clinic. On the initial evaluation at the outpatient clinic, a complete volar dislocation of the DRUJ accompanied by a fracture of the ulnar head and a volar-ulnar aspect of the distal radius fracture were detected (Fig. 1). She did not have any comorbidities, was not on any medication, and her laboratory results were unremarkable.

**Fig. 2:**
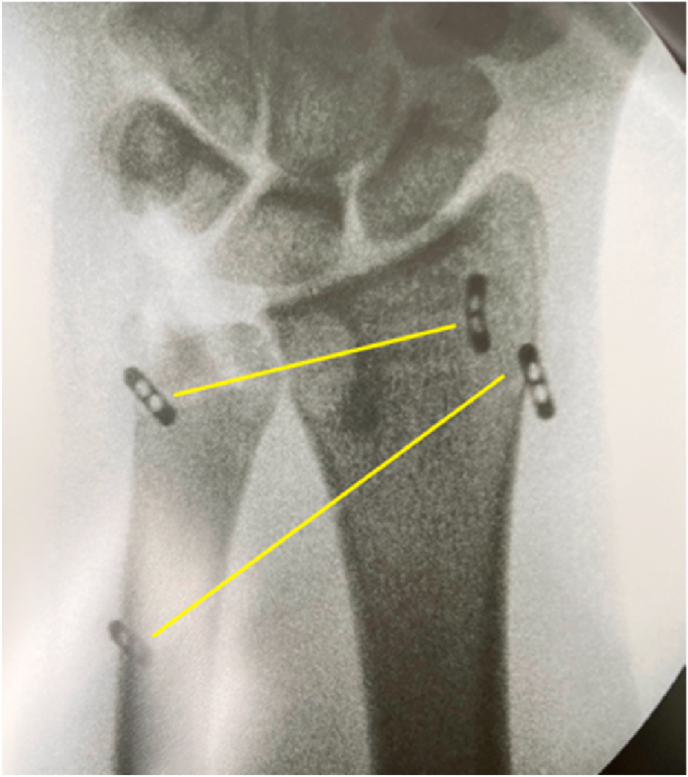
Double suture-button construct.

An orthopaedic surgeon performed the surgery and the patient understood that the operation was performed as an attempt to improve her wrist functions. As detailed by Garcia-Elias et al., a dorsal incision was made through the fifth compartment [4]. At the sigmoid notch and around the dorsal ulnar head, scar tissue was identified, although interposed structures were not observed. The DRUJ could be reduced following the excision of scar tissue at the sigmoid notch and around the dorsal ulnar head. We found 30% of the articular surface of the ulnar head was fractured, and a portion of the fracture was attached to the ECU tendon sheath. The DRUJ remained unstable in all forearm rotation positions after the decrease. The Triangular Fibrocartilage Complex (TFCC) was deemed irreparable, and the radioulnar ligament could not be repaired and distal radioulnar ligament reconstruction with palmaris longus graft could not be performed due to the possibility of ulnar head fracture during tunneling for the tendon graft tunnel. The distal oblique bundle was reconstructed utilizing the suture-button approach as described by de Vries [5]. There was still subluxation when the wrist was passively pronated and supinated despite the improvement in stability. Another suture-button plate suspension fixation was accomplished through the DRUJ, perpendicular to the long axis of the radius and ulna (Fig. 2), according to Liu, the DRUJ maintained stability in all positions [6].

The surgical site was bandaged with pads that applied pressure to maintain the functional position of the right wrist. On the first postoperative day, gentle forearm exercises including rotation, flexion, and extension were commenced. There was no splint or cast applied.

**Fig. 3:**
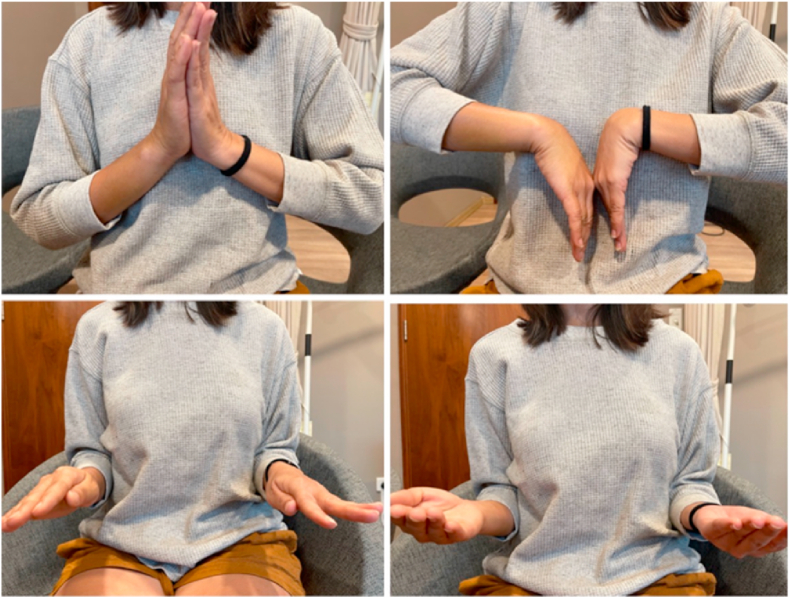
Follow up after 6 months.

The wrist and forearm's range of motion gradually and without issues regained. At two months, plain radiography confirmed a stable reduction of the DRUJ. At 6 months, she achieved 70° wrist flexion, 70° wrist extension, 80° forearm supination, and 70° forearm protonation, according to a physical examination (Fig. 3). There was no pain throughout daily activities.

## 3 Discussion

A DRUJ dislocation is an uncommon occurrence. This injury, if misdiagnosed or mismanaged, causes a total loss of pronation-supination, resulting in a severe functional impairment [1]. Volar DRUJ dislocation is an uncommon injury. It was first described in 1972 by Dameron [7]. In as many as 50% of previously documented cases, it was overlooked. Extreme supination of the forearm on a fixed hand [[7], [8], [9]], because of a simple fall [7], and weight-lifting [6] can produce volar dislocation [9].

Distal radioulnar joint disruption occurs in four basic patterns: anterior type, produced by hypersupination; posterior type, induced by hyperpronation; vertical type, frequently caused by trauma combined with radial fracture or growth disruption; and rotatory type, caused by malunited fracture [2]. The majority of papers on this topic discuss patients diagnosed and treated in an acute situation. This makes the described case even less common.

A number of techniques for stabilizing the DRUJ have been described; Adams et al. [10], described anatomical reconstruction of the radioulnar ligament using palmaris tendon graft, Riggenbach et al. [11] repaired the DOB with an extensor indicis proprius tendon graft, Stein et al. [12] present a modified Adams approach using a tendon transplant with polyester suture, Brink et al. [13] inserted a palmaris longus tendon graft at the site of the DOB between the radius and ulna.

In our situation, the chosen treatment (open reduction, TFCC reattachment, and dorsal plicature) is similar to that of previous authors [1,2]. However, the TFCC was completely detached and could not be reattached; furthermore, reconstruction with tendon graft to restore stability carries the risk of fracturing the ulnar head during tunneling for tendon graft passage.

de Vries et al. [5] describe a minimally invasive technique for distal radioulnar joint stability employing a suture-button construct implanted percutaneously in the direction of the distal oblique bundle in the distal interosseous membrane. It was shown significantly reduced post-traumatic DRUJ instability. In our situation, however, once the suture-button was tightened and DRUJ stability was checked, the ballottement was still positive and there remained subluxation of the ulnar head, so we decided to add a second suture-button construct, as described by Liu et al. [6], to improve stability. After the placement of the second suture button. The DRUJ motion was smooth and had a smooth range of motion. Because the technique permits early functional exercise, non-weight-bearing forearm activities, including rotation, flexion, and extension, were initiated.

In our situation, we believe that reconstructing the DOB alone was insufficient to establish stability. This may be due to the loss of numerous stabilizing structures of the DRUJ, as well as a degree of contracture around the forearm, which hindered stability during DRUJ motion. This double structure replicates the DOB and distal radioulnar ligament functions.

## 4 Conclusion

In conclusion, this case report describes a patient with irreducible chronic volar dislocation of the DRUJ, and repair of the distal oblique bundle alone may not be adequate to achieve stable DRUJ reduction. To prevent DRUJ instability or dislocation following surgery, the DRUJ should be assessed for stability immediately after distal oblique bundle reconstruction utilizing suture-button. The double distal radioulnar reconstruction restored the DRUJ's stability and yielded a satisfactory functional outcome.

## Sources of funding

There was no funding

## Availability of data and material

All data will be provided upon request.

## Conflicts of interest

The authors declare that they have no competing interests.

## Ethical approval

None.

## Consent

Written informed consent was obtained from the patient for publication of this case report and accompanying images. A copy of the written consent is available for review by the Editor-in-Chief of this journal on request.

## Author contribution

All authors co-wrote the paper and discussed the results for the manuscript preparation. All authors have read and approved the final manuscript.

## Guarantor

Diaz Novera.
